# Optimizing implementation: elucidating the role of behavior change techniques and corresponding strategies on determinants and implementation performance: a cross-sectional study

**DOI:** 10.1186/s43058-024-00604-w

**Published:** 2024-06-20

**Authors:** Eveline M. Dubbeldeman, Mathilde R. Crone, Jessica Christina Kiefte-de Jong, Rianne M.J.J. van der Kleij

**Affiliations:** 1 Department of Public Health and Primary Care, Leiden University Medical Center, Leiden, the Netherlands; 2 Department of Health Promotion, Maastricht University, Maastricht, the Netherlands; 3 Department of Public Health and Primary Care/LUMC-Campus The Hague, Leiden University Medical Center, The Hague, the Netherlands

**Keywords:** Implementation, Behavior change techniques, Strategies, Guidelines, Youth care, Practice

## Abstract

**Introduction:**

Behavior change techniques (BCTs) are considered as active components of implementation strategies, influencing determinants and, ultimately, implementation performance. In our previous Delphi study, experts formulated ‘implementation hypotheses’, detailing how specific combinations of BCTs and strategies (referred to as BCT-strategy combinations) might influence determinants and guideline implementation within youth care. For example, educational meetings providing instructions on guideline use were hypothesized to enhance practitioners' knowledge and, consequently, guideline implementation. However, these hypotheses have not been verified in practice yet.

**Method:**

We conducted a cross-sectional study involving practitioners and management professionals from youth (health)care organizations. Using questionnaires, we obtained data on the presence of BCT-strategy combinations and their perceived influence on determinants and implementation performance. Chi-squared tests and regression analyses were employed to determine the influence of specific BCT-strategy combinations on determinants and implementation performance.

**Results:**

Our analyses included data from 104 practitioners and 34 management professionals. Most of the management professionals indicated that the BCT-strategy combinations positively influenced or had the potential to influence their implementation performance. At the practitioner level, half of the combinations were perceived to have a positive influence on determinants and implementation performance. Furthermore, practitioners who reported the absence of BCT-strategy combinations were more skeptical about their potential influence on determinants and implementation performance.

**Conclusion:**

Several BCT-strategy combinations were perceived to improve or potentially improve implementation performance of both practitioners and management professionals. In the development and evaluation of implementation efforts, we advocate for clearly describing the implementation effort’s objective and using frameworks that detail the BCTs inducing behavior change, the strategy employed, and the processes driving the observed changes. Understanding these interconnected processes is important in designing targeted, evidence-based behavior change interventions. This understanding optimizes resource allocation and contributes to the overall success of implementation efforts in youth care.

**Supplementary Information:**

The online version contains supplementary material available at 10.1186/s43058-024-00604-w.

Contributions to the literature

- Implementation research often investigates the effectiveness of implementation strategies in addressing determinants but fails to describe which behaviour change components are responsible for the observed outcomes.
- We explored the interconnected process between determinants, behavior change techniques, strategies, and implementation performance in Dutch youth care, providing unique insights into this complex relationship.
- Our study emphasize the need for a nuanced understanding of the processes influencing implementation outcomes.

## Introduction

In Dutch youth care, numerous evidence-based guidelines and interventions exist to support the identification and/or management of child psychosocial problems, child abuse and neglect, parenting problems, and to support parents with mental health problems (further referred to as youth care guidelines) [1]. However, the availability of evidence-based guidelines does not guarantee their optimal implementation in practice [2–5]. Implementation is defined as: ‘the degree to which settings and staff members deliver a program or apply a policy as intended’ [6]. The implementation of guidelines poses inherent challenges, particularly within the realm of youth care. The interdisciplinary nature of the field, the requirement to address sensitive topics with vulnerable families, and the additional pressure stemming from growing waiting lists, increased administrative burdens, and a persistent personnel shortage all contribute to the increased difficulty of effective implementation in youth care [7]. Hence, research is increasingly focused on the implementation of guidelines and interventions. Various theoretical frameworks supporting the implementation process have been developed. These frameworks focus on determinants influencing implementation [8–10] and provide taxonomies for effective implementation strategies [11]. Implementation determinants are factors that can either facilitate or hinder successful implementation. These determinants may pertain to the innovation itself (e.g., complexity), the individuals involved (e.g., capability), the (external) organizational context (e.g., partnership & connection and funding), and the implementation process (e.g., engaging) [8]. Implementation strategies, including educational outreach, learning collaboratives, and the use of opinion leaders, are methods to address these determinants and optimize the implementation of innovations [11].

However, the influence of specific implementation strategies on determinants and how this interaction contributes to either implementation success or failure remains unclear. For example, several implementation strategies have been considered effective to change skills, such as educational outreach visits, learning collaboratives, and educational meetings [12]. Yet, the effectiveness of these strategies is not solely dependent on their direct influence on determinants; other critical components play pivotal roles in shaping the outcomes of implementation strategies [13, 14]. Behavior Change Techniques (BCTs), as active components embedded within strategies, serve as specific techniques designed to induce behavior change. Examples of BCTs include providing instructions on how to perform behaviors, action planning, and using prompts or cues [15]. For instance, employing educational meetings as a strategy to address lack of knowledge on guideline use may provide information. However, the specific BCT of providing instructions on guideline use during these meetings may truly influence practitioners' knowledge. The relationship between BCTs, strategies, and their influence on determinants and, in turn, practitioners’ performance in guideline implementation, highlights the interconnectedness of these elements within the implementation process. Exploring and understanding these relationships facilitates designing effective, tailored, and evidence-based behavior change interventions, optimizing resource allocation, and ultimately improving the success of implementation efforts [16]. There is a need for a clear understanding of how strategies and BCTs collectively influence determinants and, consequently, impact implementation performance [17].

### Delphi study

In a previous four-round Delphi study [18], we asked implementation experts to 1) identify important and changeable (i.e., relevant) determinants of youth care guideline implementation and 2) formulate feasible and potentially effective ‘implementation hypotheses’ for the relevant determinants. Building on the work by French et al. [19], we used the term ‘implementation hypotheses’ to detail how implementation determinants, and in turn, implementation performance might be influenced by specific BCTs and implementation strategies (i.e., BCT-strategy combination). In the first round, experts identified relevant determinants through closed-ended questions, including a preselected list of 44 determinants informed by a systematic review [20] and non-published data on a Dutch youth care guideline. In the second round, experts were tasked with formulating implementation hypotheses. Informed by existing literature on links between determinants, behaviour change, and strategies [12, 21], experts were provided a preselected list of BCTs and implementation strategies for each relevant determinant to formulate implementation hypotheses. The subsequent round focused on reviewing, finalizing, and rating these implementation hypotheses. Each expert had the opportunity to reassess and finalize their choices based on the anonymous rationales provided by all participants. Employing a ranking-type Delphi with fixed-sum questions, experts were asked to allocate 100 points to all formulated hypotheses for each determinant.

**Table:** 

The implementation process involves various components. To assist readers in navigating the text, we used abbreviations that refer to these specific components, which are outlined below:	
**D**	Determinant
**BCT**	Behavior Change Technique
**S**	Implementation strategy
**IOD**	Influence on Determinant
**O**	Outcome (implementation performance)

**Fig. 1:**
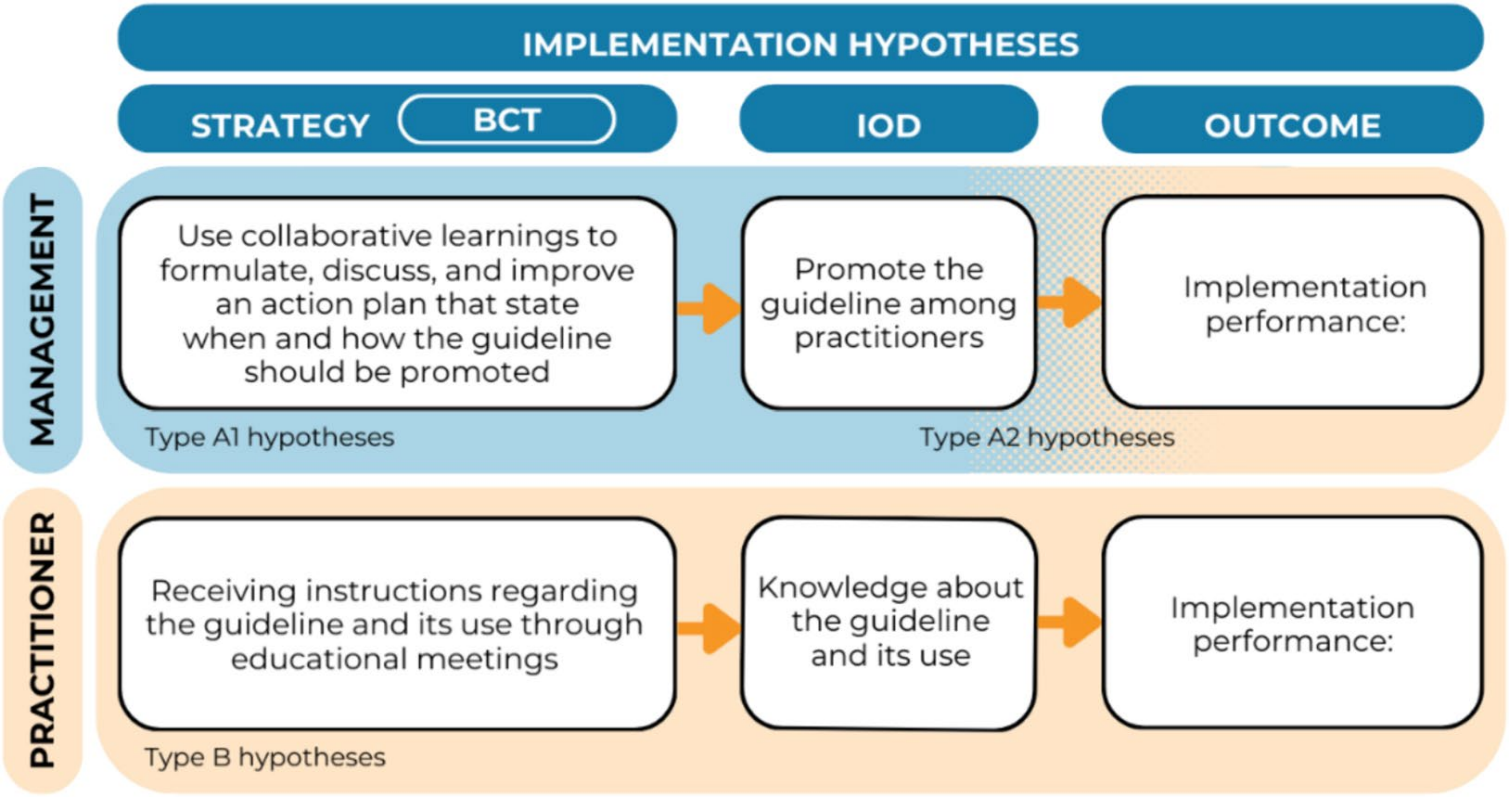
Implementation hypotheses. BCT = behavior change technique; IOD = influence on determinant

Our Delphi study revealed that experts considered determinants relating to the process of implementation (i.e., guideline promotion, mandatory education, presence of a motivated implementation leader, and management support) and knowledge and skills (i.e., guideline knowledge and communication skills) relevant for the implementation of youth care guidelines. Moreover, the Delphi study yielded the formulation of two distinct types of hypotheses: type A hypotheses and type B hypotheses, visualized in Fig. 1. Type A hypotheses were formulated for the management professionals representing management and/or policy makers responsible for facilitating guideline implementation in the organization (further referred to as management professionals). These hypotheses describe how specific strategies, including BCTs, can influence implementation strategy performance (i.e., type A1 hypotheses), which in turn may influence practitioners’ implementation performance (i.e., type A2 hypotheses). For example, to implement a new guideline, it is important that this guideline is promoted among practitioners [D]. It is hypothesized that the use of an action plan [BCT] -formulated, discussed, and improved during collaborative learnings [S]- will facilitate guideline promotion by management professionals [IOD]. Guideline promotion may, in turn, influence practitioners’ guideline implementation in practice [O]. Type B hypotheses are focused on the level of the practitioner applying the guideline in practice. Addressing a lack of knowledge regarding guideline use [D] may involve receiving instructions [BCT] through educational meetings [S], which is expected to increases their knowledge on guideline use [IOD]. This, in turn, may improve practitioners’ implementation performance [O].

Logically, implementation strategies can encompass various BCTs, and vice versa. For example, learning collaboratives [S] can be applied to provide instructions [BCT] but also to provide feedback on behaviors [BCT], while providing instructions can also be done via educational meetings [S]. In the Delphi study, we specifically focused on formulating hypotheses including one BCT with one implementation strategy, aiming for a systematic evaluation of each 1–1 combination. By concentrating on these core components, we aimed to provide a clear understanding of the interconnectedness of the identified BCT-strategy combination and their influence on the determinant and, in turn, implementation performance. In total, 46 hypotheses were formulated ranging from six to nine different hypotheses per determinant. However, these hypotheses have not been verified in practice yet.

As previously emphasized, understanding how specific determinants are influenced by BCTs and implementation strategies is important for optimizing implementation efforts. Therefore, the current study represents as a critical step in evaluating the validity of the hypotheses proposed in our earlier Delphi study within the real-world context of youth care. Specifically, we assessed whether the hypothesized BCT-strategy combinations were associated with a change in the linked determinants, and in turn, self-reported implementation performance. We focused on the top-ranking two hypotheses for each determinant based on expert ratings in the Delphi study (Table 1).

**Table 1:** Implementation hypotheses assessed in this study

	**Determinant**	**Behaviour Change Technique**	**Strategy**
**PROCESS OF IMPLEMENTATION**	Guideline promotion	Action planning	Create a learning collaborative
		Prompts/cues	Conduct educational meetings
	Mandatory education	Action planning	Assess for readiness and identify barriers and facilitators
		Action planning	Conduct local needs assessment
	Presence of a motivated implementation leader	Social support (practical)	Provide ongoing consultation
		Social comparison	Recruit, designate and train for leadership
	Management support	Social support (practical)	Obtain formal commitments
		Social support (practical)	Conduct local consensus discussions
**KNOWLEDGE** **AND SKILLS**	Knowledge on guideline use	Instructions on how to perform a behaviour	Create a learning collaborative
		Instructions on how to perform a behaviour	Conduct educational meetings
	Communication skills	Behavioural practice/rehearsal	Conduct educational outreach visits
		Behavioural practice/rehearsal	Conduct ongoing training

#### Methods

To explore whether the expected changes, as predicted by the hypotheses, could be verified in practice, we conducted a cross-sectional study. An online questionnaire was developed using the web-based survey tool Qualtrics [22] and was distributed between March and October 2021. Reporting follows the STROBE guidelines (Additional File 1) [23].

### Participants and procedures

The implementation hypotheses to be explored (Table 1) were related to two groups of professionals within youth (health) care, namely management professionals and practitioners. Therefore, employees affiliated with either of these groups where eligible to participate. To ensure a diverse and representative study population, we purposely sampled organizations in four regions with distinct levels of urbanization: Amsterdam, Haaglanden, Friesland, and Brabant. Organizations in which youth care guidelines [1] were implemented (i.e., youth health care, municipal health services, well-baby clinics, and mental health care), were contacted by phone. Interested organizations received an email containing an information letter and questionnaire link, which was then disseminated to eligible participants. Prior to participation, digital informed consent was obtained from all respondents.

### Implementation hypotheses

As previously detailed, the Delphi study introduced two types of hypotheses: type A for management professionals, and type B for practitioners. We verified two type A hypotheses for each of the following implementation determinants: promotion of the guideline, mandatory education, motivated implementation leader, and management support (*n* = 8). Two type B hypotheses were formulated for ‘knowledge about the guideline’ and ‘communication skills’ (*n* = 4). In Additional File 2, all 12 implementation hypotheses are described in more detail.

### Questionnaire

First, respondents were queried about their occupational roles (i.e., management professional or practitioner) and general characteristics such as the type of organization, professional function, years of experience, and working hours. Management professionals were asked whether their organization implemented youth care guidelines, while practitioners were questioned about their use of these guidelines in their daily functions. If affirmative, participants were asked to specify the two most used guidelines. Subsequently, participants were represented with the complete questionnaire for each reported guideline. Practitioners were prompted to rate their current guideline implementation performance (further referred to as self-reported implementation performance) on a 5-point Likert scale, ranging from extremely bad (1) to extremely good (5). The questionnaire featured a clear definition of implementation performance: 'Applying the recommendations, advice, and/or action instructions in practice as intended.' An illustrative example was provided for clarification: 'In the context of the reporting code, implementation performance involves going through the five steps before the professional decides whether to report to Child Protective Services.' Practitioners were instructed to base their ratings on their experiences at the time of completing the questionnaire, reflecting the current moment. Then, based on participants’ occupational roles, we obtained data regarding whether specific BCTs and implementation strategies were employed, and their perceived influence on determinants and, consequently, implementation performance.

### Type A hypotheses

To verify type A1 hypotheses in practice, we asked management professionals about whether specific BCT-strategies combinations were performed (e.g., ‘Is there any concrete action plan formulated [BCT] that state when and how to promote the guideline among practitioners?’ and ‘Are learning collaboratives [S] organized in which this action plan is discussed and improved?’). Furthermore, we asked about whether the BCT-strategy combination has facilitated a change in the determinant (e.g., ‘An action plan [BCT], -formulated during collaborative learnings [S]- helps me to promote the guideline among practitioners [IOD]’). To assess whether type A1 hypotheses influenced practitioners implementation in practice (type A2 hypotheses), we assessed practitioners’ experience on implementation determinants (e.g., ‘I am experiencing that the guideline is promoted within the organization [D]’) and their influence on guideline implementation (e.g., ‘Promotion of the guideline could/has … my actual guideline use [O]’).

### Type B hypotheses

Since type B hypotheses are focused on the level of practitioners, we asked practitioners about the presence of BCT-strategy combinations (e.g., ‘Did you receive instructions [BCT] on the guideline and its use?’ and ‘Did you receive specific instructions on guideline use during educational meetings [S]’). We also assessed the influence of the BCT-strategy combination on the implementation determinant (i.e., ‘Receiving specific instructions about guideline use helps me to increase my knowledge regarding guideline use [IOD]’). Finally, we asked practitioners about the influence of the implementation determinant on their guideline implementation (i.e., ‘Increasing my knowledge regarding guideline use has … my actual guideline use [O]’).

Participants were provided with three possible responses regarding the execution of implementation strategies and BCTs: 'yes', 'no', and 'I don’t know'. Questions concerning the IODs were rated on a 5-point Likert scale ranging from 1 (totally disagree) to 5 (totally agree). The influence on implementation performance was rated on a 5-point Likert scale ranging from 1 (totally deteriorated) to 5 (totally improved).

A complete overview of the questions is presented in Additional File 3. In instances where participants reported certain implementation strategies or BCTs were not performed or did not influence implementation determinants, they were redirected to a hypothetical version of the same question (e.g., ‘An action plan [BCT] -formulated during collaborative learnings [S]- could help me to promote the guideline among practitioners [O]’). This enabled us to assess participants’ perceptions of the potential influence of these aspects on implementation performance.

### Statistical analyses

We excluded participants who: 1) did not sign the informed consent, 2) did not complete the questionnaire, or 3) did not report to use any guidelines. After data reviewing, no missing data or outliers were found. To discern between the presence or absence of specific components within hypotheses, we converted categorical responses (i.e., no/I don’t know = no(0); yes = yes(1)) and Likert-scale responses (1,2,3 = no(0); 4,5 = yes(1)) into dichotomous variables Based on these responses, a new variable was created, termed ‘implementation hypotheses part 1’. We considered the implementation hypotheses part 1 as present (coded as 1) when the variables concerning the BCTs and strategies were rated as 1. For all other combinations, the hypothesis was considered absent (coded as 0). In this study, we specifically evaluated the hypotheses associated with determinants identified in the Delphi study that -for the practitioners in our study- had a substantial impact on the implementation of youth care guidelines. Therefore, we first conducted a univariate regression analysis for each determinant to assess their influence on practitioners’ self-reported implementation performance. Determinants showing significance were further analysed to explore implementation hypotheses.

### Type A hypotheses

To evaluate type A1 hypotheses (i.e., the influence of implementation hypothesis part 1 [BCT, S] on a change in the determinant [IOD]), we used descriptive statistics. We employed a chi-square test to compare the perceived (potential) influence on implementation performance in the groups that did and did not received a specific BCT-strategy combination. Type A2 hypotheses (e.g., the influence of practitioners’ perceived management support [D] on their self-reported implementation performance [O]) were assessed using univariate and multivariate logistic regression analyses, including organization type, occupational function/profession, years of experience, working hours, and type of youth care guideline as covariates.

### Type B hypotheses

To assess type B hypotheses, we created another variable, termed ‘implementation hypotheses part 2’. We considered the implementation hypotheses part 2 as present ( coded as 1) when implementation hypothesis part 1 as well as the influence of part 1 on the implementation determinant [IOD] were rated as 1. For all other combinations, the hypothesis was considered absent (coded as 0). We used descriptive statistics and univariate and multivariate logistic regression analysis to assess the influence of implementation hypothesis part 2 [BCT, S, IOD] on practitioners’ self-reported implementation performance [O]. Covariates included in the multivariate regression analyses were organization type, occupational function/profession, years of experience, working hours, and type of youth care guideline.

We used IBM SPSS Statistics for Windows, version 25 analyse the data, with p-values below 0.05considered significant.

## Results

### Participants

#### Practitioners

In total, 148 practitioners responded to the questionnaire. Four practitioners did not sign the informed consent form, 25 did not use any guidelines of interest, and fifteen did not fully complete the questionnaire. Of the remaining 104 responses, 76 completed the questionnaire for two types of guidelines and 28 for only one guideline, which resulted in 180 unique cases eligible for analysis (Table 2).

**Table 2:** General characteristics of management professionals (*n* = 34) and practitioners (*n* = 104)

	Management *n*(%)	Practitioners *n*(%)
Organization		
*Mental health care*	21(61.8)	76(73.1)
*Forensic mental health care*	5(14.7)	11(10.6)
*Youth health care*	3(8.8)	9(8.7)
*Youth care*	1(2.9)	7(6.7)
*Other*	4(11.8)	1(1.0)
Profession [based on educational level]	NA	
*University postgraduate degree with specialization*		7(6.7)
*University postgraduate degree*		35(33.7)
*University degree*		18(17.3)
*University of applied science postgraduate degree*		44(42.3)
Experience [in years]	NA	17.76(10.05)^b^
Work hours [per week]	NA	28.88(7.14)^b^
Guideline objective^a^		
*Reporting Code Act Domestic Violence & Child Abuse*	18(31.8)	63(35.0)
*Child Check*	14(24.1)	46(25.6)
*Psychosocial*	11(19.0)	40(22.2)
*Child problems*	9(15.5)	18(10.0)
*Other*	6(10.3)	13(7.2)
Self-reported implementation performance	NA	3.85(0.64)^b^

*NA* not applicable

^a^for management *n* = 58, for practitioners *n* = 180

^b^numbers in M(sd)

### Management

In total, 49 employees with a management function responded to the questionnaire. Five responders did not sign the informed consent, three reported that guidelines of interest were not used, and seven did not fully complete the questionnaire. Of the remaining 34 responses, 24 completed the questionnaire for two guidelines and ten for only one, resulting in 58 cases eligible for analysis. Table 2 provides the general characteristics of the responders.

The univariate regression analysis showed that mandatory education was not related to practitioners’ self-reported implementation performance (Additional File 4, Table 1). Therefore, we did not evaluate the hypothesis for this determinant.

#### Implementation hypotheses type A

### Guideline promotion

**Fig. 2:**
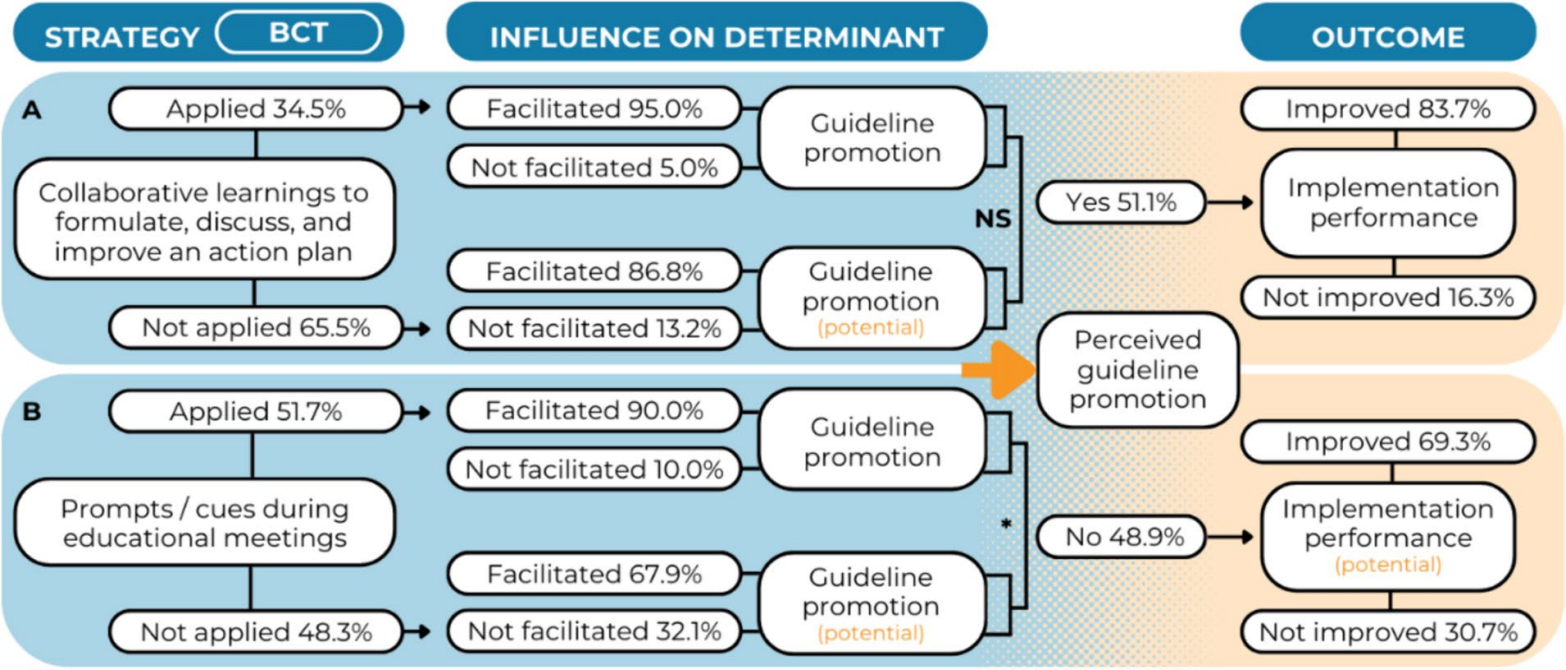
Results for the BCT-strategy combination *action planning – create a learning collaborative* **A** and *prompts/cues – conduct educational meetings* **B** regarding guideline promotion. * = significant at *p* < .05.; NS = not significant

Approximately one-third of the managers (34.5%) formulated an action plan [BCT] during collaborative learnings [S] (Fig. 2A). Among them, 95.0% responded that this strategy improved their guideline promotion within the organization [IOD]. Most of the managers who did not formulate an action plan during collaborative learnings hypothesized that this strategy could improve their guideline promotion (86.8%). No significant difference was found between these groups, as indicated by the Fisher’s Exact test (*p* = 0.653).

About half of the managers (51.7%) received prompts/cues [BCT] during educational meetings [S] (Fig. 2B). Among them, the majority (90.0%) responded that this strategy improved their guideline promotion within the organization [IOD]. About two-third of the managers who did not receive prompts/cues during educational meetings hypothesized that this strategy could improve their guideline promotion (67.9%). The difference between these groups was significant (χ^2^(1,58) = 4.33, *p* = 0.038).

Furthermore, the regression analysis revealed a significant influence of perceived guideline promotion on practitioners’ self-reported implementation performance (OR = 2.91, 95% CI [1.28–5.64], *p* = 0.009). The multivariate regression model was significant (Nagelkerke R^2^ = 0.16, *p* = 0.018) (Additional File 4, Table 2).

### Motivated implementation leader

**Fig. 3:**
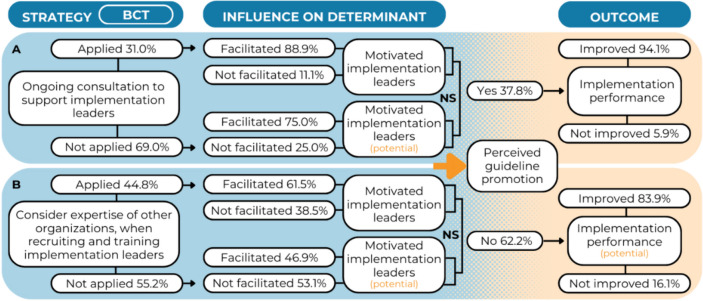
Results for the BCT-strategy combination *social support (practical) – provide ongoing consultation* **A** and *social comparison – recruit, designate, and train for leadership* **B** regarding the presence of a motivated implementation leader. * = significant at *p* < .05.; NS = not significant

Results show that 31.0% of the managers supported [BCT] implementation leaders by applying ongoing consultations [S] (Fig. 3A). Among them, 88.9% responded that this strategy facilitated managers to keep implementation leaders motivated [IOD]. Three-quarter of the managers who did not have ongoing consultation with implementation leaders, hypothesized that this strategy could facilitate managers to keep implementation leaders motivated (75.0%). The Fisher’s Exact test showed no significant difference between these groups (*p* = 0.300).

About half of the managers (44.8%) considered other organizations' expertise [BCT] while recruiting implementation leaders [S] (Fig. 3B). Among them, 61.5% responded that this strategy facilitated the recruitment of motivated implementation leaders [IOD]. Of those who did not consider the expertise of other organizations, while recruiting implementation leaders, 46.9% hypothesized that this strategy could facilitate the recruitment of motivated implementation leaders. The difference between the groups was not significant (χ^2^(1,58) = 1.24, *p* = 0.266).

Furthermore, results showed that the presence of a motivated implementation leader significantly influenced practitioners’ self-reported implementation performance, compared to the absence of a (motivated) implementation leader (OR = 4.44, 95% CI [1.67–11.84, *p* = 0.003]). The multivariate regression model was significant (Nagelkerke R^2^ = 0.19, *p* = 0.006) (Additional File 4, Table A3).

### Management support

**Fig. 4:**
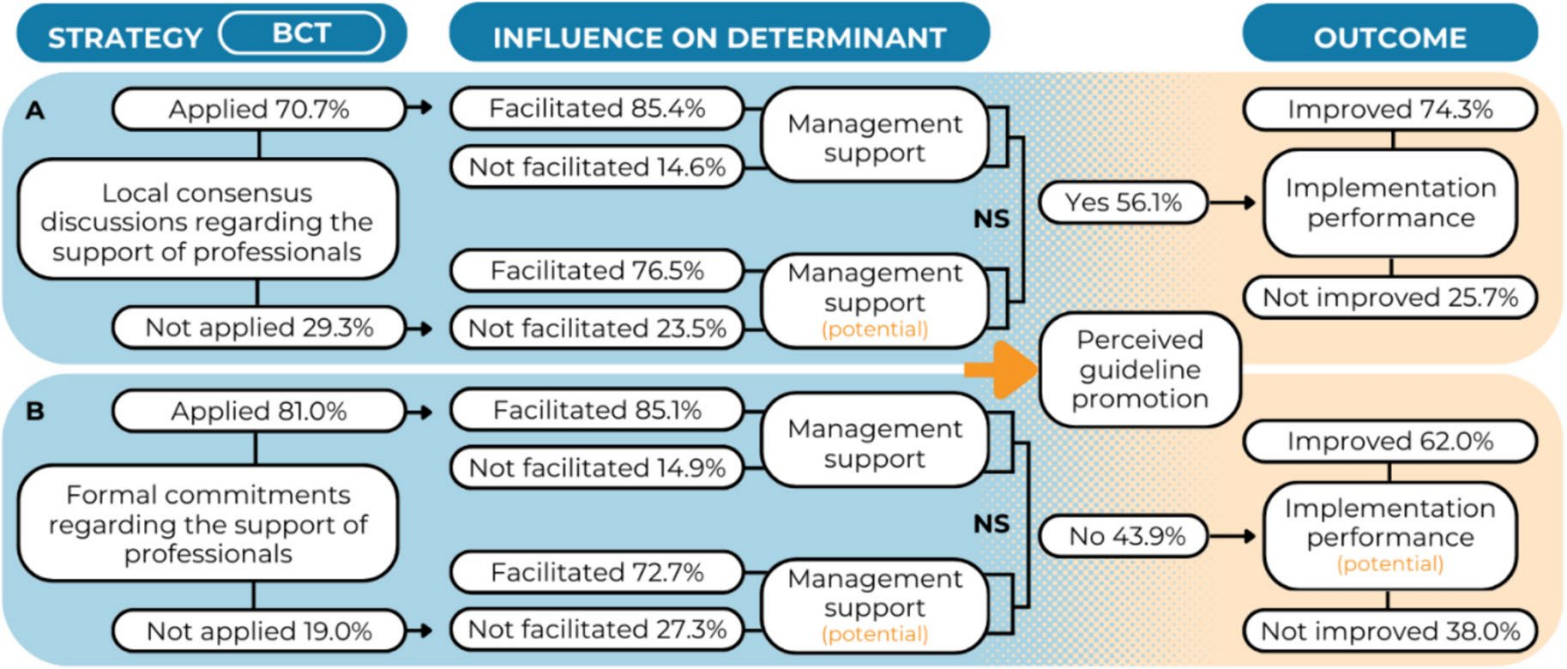
Results for the BCT-strategy combination *social support (practical) – obtain formal commitments* **A** and *social support (practical) – conduct local consensus discussions* **B** regarding management support. * = significant at *p* < .05.; NS = not significant

Results showed that 70.7% of the managers conducted local consensus discussions within their team [S] to determine how and when the guideline should be used and how to support practitioners in this process [BCT] (Fig. 4A). Among them, 85.4% responded that this strategy improved their actual support towards practitioners [IOD]. Most of the managers who did not conduct local consensus discussions hypothesized that this strategy could improve their support towards practitioners (76.5%). The Fisher’s Exact test showed no significant difference between these groups (*p* = 0.458).

Eighty-one percent of the managers obtained formal commitments within their team [S] that state how and when the guideline should be used by practitioners and their commitment to support them in this process [BCT] (Fig. 4B). Among them, the majority (85.1%) responded that this strategy improved their actual support towards practitioners [IOD]. Among managers who did not obtain formal commitments, 72.2% hypothesized that this strategy could improve their support towards practitioners. The Fisher’s Exact test showed no significant difference between these groups (*p* = 0.381).

Furthermore, the regression analysis showed that perceived management support significantly influenced practitioners’ self-reported implementation performance (OR = 4.44, 95% CI [1.95–10.11], *p* < 0.001). The multivariate regression model was significant (Nagelkerke R^2^ = 0.21, *p* = 0.002) (Additional File 4, Table A4).

#### Implementation hypotheses type B

### Knowledge on guideline use

**Fig. 5:**
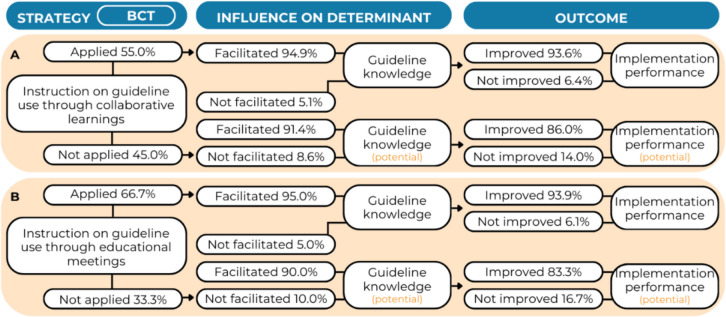
Results for the BCT-strategy combination *instructions on how to perform a behaviour – create a learning collaborative* **A** and *instructions on how to perform a behaviour – conduct educational meetings* **B** regarding knowledge on guideline use

About half of the practitioners (55.0%) received instructions on guideline use [BCT] through collaborative learnings [S] (Fig. 5A). Among them, 94.9% responded that this strategy improved their knowledge [IOD] and increased guideline implementation [O] in 93.6% of these cases. The majority of practitioners (86.0%), who either did not receive instructions on guideline use through collaborative learning sessions or reported that this strategy did not enhance their knowledge, hypothesized that an increase in knowledge through this strategy could lead to improved guideline implementation. The regression analysis showed that increased guideline knowledge after receiving instructions through collaborative learnings did not significantly influenced practitioners’ self-reported implementation performance (OR = 2.19, 95% CI [1.00–4.77], *p* = 0.050) (Additional File 4, Table A5).

Two-third of the practitioners (66.7%) were provided instructions on guideline use [BCT] through educational meetings [S] (Fig. 5B). Among them, 95.0% responded that this improved their knowledge [IOD], which, in turn, increased guideline implementation [O] in 93.9% of the cases. The majority of practitioners (83.3%), who either did not receive instructions through educational meetings or stated this strategy did not improve their knowledge, hypothesized that enhanced guideline knowledge through educational meetings could lead to improved guideline implementation. The regression analysis showed that increased guideline knowledge after receiving instructions through educational meetings significantly influenced practitioners’ self-reported implementation performance (OR = 2.22, 95% CI [1.03–4.79], *p* = 0.042). The multivariate regression model was significant (adjusted R^2^ = 0.14, *p* = 0.048) (Additional File 4, Table A6).

### Communication skills

**Fig. 6:**
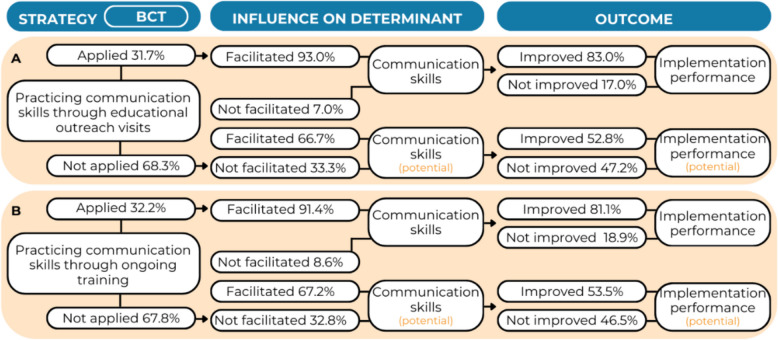
Results for the hypotheses *behavioral practice/rehearsal – conduct educational outreach visits* **A** and *behavioral practice/rehearsal – conduct ongoing training* **B** regarding communication skills

About one-third of the practitioners (31.7%) practiced communication skills [BCT] during educational outreach visits [S] (Fig. 6A). Among them, 93.0% responded that this strategy improved their communication skills [IOD] and, consequently, increased guideline implementation [O] in 83.0% of these cases. Among practitioners who either did not practice their communication skills through educational outreach visits or stated this strategy did not improve their skills, only 52.8% hypothesized that improved communication skills could lead to improved guideline implementation. The regression analysis showed that increased communication skills after practicing their skills through educational outreach visits did not significantly influence practitioners’ self-reported implementation performance (OR = 2.13, 95% CI [0.83–5.47], *p* = 0.117, Additional File 4, Table A7).

About one-third of the practitioners (32.2%) practiced their communication skills through ongoing training [S] (Fig. 6B). Among them, 91.4% responded that this strategy improved their communication skills [IOD], which, in turn, increased guideline implementation [O] in 81.1% of the cases. Among practitioners who did not practice their skills through ongoing training or stated this strategy did not improve their skills, only 53.5% hypothesized that improved communication skills could lead to improved guideline implementation. The regression analysis showed that increased communication skills after practicing their skills through ongoing training significantly influenced practitioners’ self-reported implementation performance (OR = 2.80, 95% CI [1.08–7.25], *p* = 0.034). The multivariate regression model was significant (Nagelkerke R^2^ = 0.15, *p* = 0.036, Additional File 4, Table A8).

## Discussion

In the context of youth care, our study aimed to verify the previously formulated hypotheses [18] about the influence of BCT-strategy combinations on determinants and self-reported implementation performance in real-world settings. The majority of management professionals and practitioners reported positive influences when BCT-strategy combinations were employed, indicating improvements in determinants and/or implementation performance. Furthermore, even among the management professionals who reported that BCT-strategy combinations were not performed, there was still a tendency towards perceiving potential positive influences. However, practitioners tended to be more skeptical about the potential influence of BCT-strategy combinations.

Regarding guideline knowledge [D], we hypothesized that knowledge level is increased [IOD] by receiving instructions [BCT] through collaborative learnings [S], which, in turn, improves practitioners’ implementation performance [O]. In our study, 55% of the practitioners responded that receiving instructions on guideline use through collaborative learnings. Although the majority stated that receiving instructions through collaborative learnings increased their knowledge and, in turn, improved their guideline use, the multivariate regression analysis did not show a significant influence on practitioners’ self-reported implementation performance. This finding contrasts with previous research on collaborative learnings and guideline implementation in health care [24–27]. A potential explanation for this discrepancy could be our inclusion of knowledge increase as a component. Indeed, our data showed that irrespective of knowledge increase, practitioners who received instructions through collaborative learnings did indicate a significantly higher self-reported implementation performance than their counterparts. This suggests that practitioners’ knowledge might not be the key determinant causing a change in their implementation performance or practitioners in this study did not consider knowledge acquisition as a significant influence Instead, practitioners’ guideline implementation might have been influenced by the social processes that arose within collaborative learning environments [12, 28, 29]. However, the second hypothesis, proposing that providing instructions [BCT] through educational meetings [S] increases practitioner guideline knowledge [IOD] and implementation performance [O], was confirmed. These findings imply that to improve implementation performance by increasing knowledge, educational meetings might be more conducive to provide instructions than collaborative learnings. Nonetheless, considering the processes involved in behaviour change is crucial when selecting and analysing appropriate BCTs and strategies [29]. This approach goes beyond acknowledging that an interventions or strategy works by providing a comprehensive understanding of why a particular intervention or strategy is effective in specific contexts. It enables researchers and practitioners to pinpoint the specific processes contributing to observed outcomes, facilitating the refinement and customization of interventions for enhanced effectiveness [28, 29].

Regarding communication skills [D], we hypothesized that skills level is increased [IOD] by practising skills [BCT] through educational outreach visits [S], which, in turn, improves practitioners’ implementation performance [O]. However, our results did not confirm this hypothesis, also regardless of communication skills improvement. Based on these findings, there may be limited support that enhancing communication skills through practice during educational outreach visits leads to improved implementation performance within youth care. Previous research showed mixed results on the effectiveness of educational outreach visits on various implementation outcomes in health care [30–32]. More research is needed to gain more insight on the effects of educational outreach visits on skill development and guideline implementation. According to Perry and colleagues, dynamic and interactive trainings are most suitable to improve skills [33]. This is supported by previous research in the field of primary care and mental health care using standardized patients and role play. These training methods improved practitioners’ self-efficacy, skills, and confidence to use the skills in practice [34].Importantly, these results were mediated by the amount of training received [35], aligning with our results on the influence of ongoing [S] practice [BCT] on practitioners’ communication skills [IOD] and implementation performance [O].

Most of the management professionals considered action planning [BCT] in combination with learning collaboratives [S] conducive to improve guideline promotion [O]. Developing an action plan can facilitate change by providing behavioral regulation and cues, specifying when, where, and how to act [28, 29]. This, in turn, can contribute to positive workplace cultures where stakeholders take responsibility for implementation performance and quality improvement [36–38]. Additionally, even when an action plan and/or learning collaboratives were absent, most of the management still responded positively towards their potential influence guideline promotion.

Practitioners who did not receive specific BCT-strategy combinations were significantly less positive regarding their potential influence on determinants and implementation performance compared to those who did. For example, practitioners who did not receive guideline instructions [BCT] through educational meetings [S] were less likely to believe this would increase their guideline knowledge [IOD] and implementation [O] compared to those who did. This trend was consistent across nearly all hypotheses for practitioners, whereas at the management level, negative beliefs were only observed for one hypothesis. This skepticism among practitioners towards new implementation efforts could be attributed to implementation fatigue, stemming from the substantial changes experienced within the Dutch youth care system in recent years. The decentralization of the system in 2015 has led to fragmented care and increased administrative burdens. Despite the implementation of action programs, challenges such as increasing waiting lists and persistent personnel shortages persist, exacerbating the difficulties faced by practitioners. This continuous wave of changes and initiatives may have left practitioners feeling overwhelmed and fatigued. To address practitioners’ skepticism, involving them in the development of implementation strategies could be a valuable solution. By engaging practitioners, we can gain valuable insights and experiences, improving the credibility of the results and fostering a sense of ownership and responsibility. This collaborative approach is vital for optimizing implementation effects and promoting a more positive attitude towards new strategies in the youth care system [39].

### Strengths and limitations

The strength of this study is that, to our knowledge, this is the first study that explored the interconnected process between determinants, BCTs and strategies, and their influence on determinants and, in turn, implementation performance within youth care. Implementation research often investigates implementation strategies' effectiveness on a change in determinants, but fails to describe which behaviour change processes are responsible for the resulting change [40]. Developing and evaluating implementation strategies including BCTs and their MoA using, for example, the Theory of Informed Behaviour Change model [19] or the AIMD framework (Aims, Ingredients, Mechanisms, Delivery) [41], makes it possible to evaluate what core elements contribute to the effectiveness of implementation efforts [15, 40, 42, 43].

Some limitations should be noted too. Firstly, we did not include a question about managers’ self-reported implementation performance independent of the hypotheses (e.g., ‘how would you rate the extent to which the organization has promoted the guideline among practitioners?’). Consequently, we were unable to perform regression analysis for hypotheses type A1 as we did for hypotheses B. Furthermore, we could not link the data from the management to those of the practitioners. Therefore, hypotheses A1 and A2 could not be tested as one chain of hypotheses as illustrated in Fig. 2. In addition, the small sample size for the management reduced statistical power.

As highlighted in the introduction, strategies can encompass a range of BCTs, and vice versa, a single BCT may be delivered through various strategies. We acknowledge that our study's approach, which explores the relationship between individual 1–1 BCT-strategy combinations, determinants, and implementation performance, may not fully capture the complexity observed in real-world situations. It represents a deliberate simplification that allowed us to systematically assess and comprehend the impact of each specific combination. In future research, we advocate for a more nuanced exploration, considering the intricate interplay of multiple BCTs and strategies to provide a more comprehensive understanding of their interconnectedness in practical contexts.

Next, it is important to acknowledge that we did not collect data on other processes potentially influencing changes in determinants and implementation performance. There may be other psychological, physical, or social processes at play that we did not account for in our study. Exploring these processes is crucial for understanding the mechanisms behind behaviour change, thereby improving our ability to explain why interventions are effective [29].

Also, to verify the hypotheses in practice, we employed a cross-sectional method to collect data on practitioners’ and managers’ experience regarding the implementation hypotheses. However, to offer insights into the effect of BCTs and implementation strategies on implementation performance, randomized controlled trials or before-after studies (with a matched control group) are required. Nonetheless, our study provides a basis for further research on effective strategies to improve guideline implementation within youth care.

Another limitation is that the proportion of variability (R^2^) explained in the regression models were low. This indicates that, data points fall further from the regression line and thus, changes in implementation performance are only marginally explained by the dependent variable, but a significant trend between the variables is still present. On the other hand, our primary goal was to understand the relationship between the independent variables and the dependent variable and not to predict the value of the dependent variable.

Finally, since the implementation hypotheses were formulated for youth care guidelines, our results are not generalizable for other guidelines used in health care such as guidelines for cardiovascular disease or diabetes. Moreover, most of our participants were working in mental health care (61.8%) and therefore, the results may not be generalizable in other organizations in which youth care guidelines are implemented.

## Conclusions

Guideline implementation is a complex process involving determinants, BCTs, strategies, and implementation performance. By verifying our hypotheses in practice, we contributed to a better understanding of this complex relationship within youth care. According to practitioners and management professionals, several BCT-strategy combinations improved or could improve changes in determinants and/or implementation performance. Furthermore, our study underscores the need for a nuanced understanding of the processes influencing implementation outcomes.. Overall, our study provides a basis for future researchers, policy makers, and other stakeholders to develop, apply, and evaluate strategies for guideline implementation in youth care. We recommend clearly describing the implementation efforts’ objective and using frameworks that include a description of both the BCTs that will elicit behavior change, the strategy to achieve this, and the processes that drive the observed changes in implementation outcomes. Understanding the interconnected process between BCTs and strategies, and how they influence determinants and outcomes, is important for designing targeted, evidence-based behavior change interventions. Furthermore, our study illustrated that some participants, mainly practitioners, were more skeptical regarding the potential effect of implementation hypotheses. This should also be considered when developing and implementing strategies to guide the implementation process in the increasingly demanding field of youth care.

### Supplementary Information

Supplementary Material 1.

Supplementary Material 2.

Supplementary Material 3.

Supplementary Material 4.

## Data Availability

All data supporting the conclusions of this study are included in the paper and its additional files. The datasets used and/or analysed during the current study are available from the corresponding author on reasonable request.

## Abbreviations

BCT: Behavior Change Technique
D: Determinant
IOD: Influence on Determinant
O: Outcome
S: Strategy
