# Supplementary material for: Optimizing implementation: elucidating the role of behavior change techniques and corresponding strategies on determinants and implementation performance: a cross-sectional study
Source: Implement Sci Commun. 2024 Jun 20;5:68. doi: 10.1186/s43058-024-00604-w (PMC11191141; doi:10.1186/s43058-024-00604-w)
Supplement: Supplementary file 2 — Supplementary Material 2. [file 43058_2024_604_MOESM2_ESM.docx]

**Additional File 2:** Implementation hypotheses

| **Table 1.** Implementation hypotheses assessed during this study | | | |
| --- | --- | --- | --- |
|  | **Determinant** | **Behaviour Change Technique** | **Strategy** |
| **PROCESS OF IMPLEMENTATION** | Guideline promotion | Action planning | Create a learning collaborative |
|  |  | Prompts/cues | Conduct educational meetings |
|  | Mandatory education | Action planning | Assess for readiness and identify barriers and facilitators |
|  |  | Action planning | Conduct local needs assessment |
|  | Presence of a motivated implementation leader | Social support (practical) | Provide ongoing consultation |
|  |  | Social comparison | Recruit, designate and train for leadership |
|  | Management support | Social support (practical) | Obtain formal commitments |
|  |  | Social support (practical) | Conduct local consensus discussions |
| **KNOWLEDGE AND SKILLS** | Knowledge on guideline use | Instructions on how to perform a behaviour | Create a learning collaborative |
|  |  | Instructions on how to perform a behaviour | Conduct educational meetings |
|  | Communication skills | Behavioural practice/rehearsal | Conduct ongoing training |
|  |  | Behavioural practice/rehearsal | Conduct educational outreach visits |

**Type A hypotheses**

Promotion of the guideline

To promote the guideline among professionals, experts considered the following hypotheses as most effective and feasible: *1) Create a learning collaborative – Action planning*. With this hypothesis experts assumed that promotion of the guideline is facilitated by the formulation of an action plan. This plan provides the management information on when and how to promote the guideline among professionals. Collaborative learnings are recommended to formulate, discuss, and improve the content of this plan. 2) *Educational meetings – Prompts/Cues*. Experts hypothesized that prompts and cues, received during educational meetings, facilitate the management to promote the guideline among professionals.

Mandatory education

Experts considered the following hypotheses as most effective and feasible to facilitate the organization of mandatory education. 1) *Conduct local needs assessments – Action planning* and 2) *Assess readiness and identify barriers and facilitators – Action planning*. Experts hypothesized that the organization of mandatory education is facilitated by the formulation of an action plan. Prior to the formulation of this plan, experts recommended to assess what is needed to organize mandatory education and whether professionals are willing to attend mandatory education. Furthermore, barriers and facilitators to the organization of mandatory education need to be identified to facilitate the formulation of an action plan.

Motivated implementation leader

To enhance the presence of a motivated implementation leader, the following hypotheses were considered as most effective and feasible: 1) *Provide ongoing consultation – Social support (practical).* With this hypothesis, experts assumed that the management needs to support implementation leaders by providing them ongoing consultations and guidance on how to perform their task. 2) *Recruit, designate, and train for leadership – Social comparison*. Experts assumed that the assignment of an implementation leader who is motivated in performing his/her task, is facilitated by recruiting and training eligible employees. The management is advised to consider the expertise and experience from other organizations to aid them during this process.

Management support

The following hypotheses were considered by experts as most effective and feasible to enhance management support: 1) *Conduct local consensus discussions – Social support (practical)* and 2) *Obtain formal commitments – Social support (practical)*. With these hypotheses, experts assumed that professionals will benefit most when practical help is offered. Experts recommended that the management needs to plan consensus discussions about when and how the guideline should be used by professionals, which will aid the management in supporting professionals with their guideline use. Furthermore, experts assumed that formal commitments from the management that state when and how the guideline should be used by professionals and what they will do to support professionals, facilitate actual support.

**Type B hypotheses**

Knowledge about the guideline and its use

We analyzed the following hypotheses that experts considered as most effective and feasible to enhance knowledge about guideline use: 1) *Instructions on how to perform the behavior – Create a learning collaborative* and 2) *Instructions on how to perform the behavior – Conduct educational meetings.* Experts hypothesized that professionals’ knowledge will be increased by providing them information and instructions on the guideline. Collaborative learnings and educational meetings were assumed to facilitate teaching professionals about the guideline.

Communication skills

We analyzed the following hypotheses that experts considered as most effective and feasible to enhance professionals’ communications skills: 1) *Behavioral practice/rehearsal – Conduct ongoing training* and 2) *Behavioral practice/rehearsal – Conduct educational outreach visits.* Experts hypothesized that professionals will develop communication skills by practice and rehearsal. To facilitate this process, experts recommend to organize and conduct educational outreach visits. Furthermore, experts recommended to provide training in an ongoing way.
