# Supplementary material for: Optimizing implementation: elucidating the role of behavior change techniques and corresponding strategies on determinants and implementation performance: a cross-sectional study
Source: Implement Sci Commun. 2024 Jun 20;5:68. doi: 10.1186/s43058-024-00604-w (PMC11191141; doi:10.1186/s43058-024-00604-w)
Supplement: Supplementary file 3 — Supplementary Material 3. [file 43058_2024_604_MOESM3_ESM.docx]

**Additional File 3:** Overview of the survey questions per determinant and hypotheses.

**Type A hypotheses**

| PROMOTION OF THE GUIDELINE | | |
| --- | --- | --- |
|  |  |  |
| Create a learning collaborative - Action planning |  | Educational meetings - Prompts/Cues |
|  |  |  |
| Management professionals | | |
|  |  |  |
| Is there any concrete action plan formulated? |  | During educational meetings, do you receive prompts/cues (e.g. reminders) to promote the guideline among Practitioners?  the guideline? |
|  |  |  |
| Are collaborative learnings organized to discuss and improve this plan? |  | These educational meetings help me to use prompts/cues |
|  |  |  |
| Collaborative learnings helps me to formulate a concrete action plan |  | These educational meetings could help me to use prompts/cues |
|  |  |  |
| Collaborative learnings could help me to formulate a concrete action plan |  | Prompts/cues helps me to promote the guideline among practitioners. |
|  |  |  |
| An action plan helps me to promote the guidline among practitioners. |  | Prompts/cues could help me to promote the guideline among practitioners. |
|  |  |  |
| An action plan could help me to promote the guidline among practitioners. |  |  |
|  |  |  |
| Practitioners | | |
|  |  |  |
| I am experiencing that the guideline is promoted within the organization. | | |
|  |  |  |
| Promotion of the guideline has … my actual guideline use. | | |
|  | | |
| Promotion of the guideline could … my actual guideline use. | | |

| MANDATORY EDUCATION | | |
| --- | --- | --- |
|  |  |  |
| Conduct local needs assessments - Action planning |  | Assess readiness and identify barriers and facilitators - Action planning |
|  |  |  |
| Management professionals | | |
|  |  |  |
| Do you provide education on guideline use to Practitioners? | | |
|  |  |  |
| Is this education mandatory? | | |
|  |  |  |
| Is there any concrete action plan formulated that state how mandatory education is organized (which education is when provide to whom)? | | |
|  |  |  |
| Is there, during the formulation of this action plan, assessed  what is needed to organize mandatory education? |  | Is there, during the formulation of this action plan, assesed whether  Practitioners are willing to attent mandatory education? |
|  |  |  |
| Determining what is needed to organize mandatory education  helps me to formulate this action plan |  | Assessing professionals willingness to attent mandatory education helps me to formulate this action plan |
|  |  |  |
| Determining what is needed to organize mandatory education could help me to formulate this action plan |  | Assessing professionals willingness to attent mandatory mandatory education could help me to formulate this action plan |
|  |  |  |
|  |  | Is there, during the formulation of this plan, assesed whether deteminants could influence the organisation of mandatory education? |
|  |  |  |
|  |  | Identifying determinants influencing the organization of mandatory education helps me to formulate this action plan |
|  |  |  |
|  |  | Identifying determinants influencing the organization of mandatory education could help me to formulate this action plan |
|  | | |
| Such an action plan helps me to organize mandatory education. | | |
|  | | |
| An action plan could help me to organize mandatory education. | | |
|  |  |  |
| Practitioners | | |
|  |  |  |
| Did you have any education on guideline use? | | |
|  |  |  |
| Was this a mandatory education? | | |
|  | | |
| Mandatory education on guideline use has ... my actual guideline use. | | |
|  | | |
| Mandatory education on guideline use could ... my actual guideline use. | | |

| MOTIVATED IMPLEMENTATION LEADER | | |
| --- | --- | --- |
|  |  |  |
| Provide ongoing consultation - Social support (practical) |  | Recruit, designate and train for leadership - Social comparison |
|  |  |  |
| Management professionals | | |
|  |  |  |
| Does your organization have an implementation leader? | | |
|  |  |  |
| Do you regularly consult implementation leaders to support  them in the performance of their function? |  | Do you consider expertise and experiences of other organizations,  while recruiting and training implementation leaders? |
|  |  |  |
| Supporting implementation leaders helps me to keep them motivated. |  | The expertise and experiences of other organizations help me in the recruitement and training of motivated implementation leaders |
|  |  |  |
| Such consults could help me to support implementation leaders |  | The expertise and experiences of other organizations could help me in the recruitement and training of motivated implementation leaders |
|  |  |  |
| Supporting implementation leaders helps me to keep them motivated. |  |  |
|  |  |  |
| Supporting implementation leaders could help me to keep them motivated |  |  |
|  |  |  |
| Practitioners | | |
|  |  |  |
| Does your organization have an implementation leader? | | |
|  |  |  |
| I am experiencing that the implementation leader is motivated in performing his/her task. | | |
|  | | |
| The presence of a motivated implementation leader has ... my actual guideline use. | | |
|  | | |
| The presence of a motivated implementation leader could ... my actual guideline use. | | |

| MANAGEMENT SUPPORT | | |  |
| --- | --- | --- | --- |
|  |  |  |  |
| Conduct local consensus discussions - Social support (practical) |  | Obtain formal commitments - Social support (practical) |  |
|  |  |  |  |
| Management professionals | | |  |
|  |  |  |  |
| Are consensus discussions planned about how and when  the guideline should be used by practitioners and how to support them? |  | Are formal commitments obtained on how and when  the guideline should be used by practitioners and their commitment? |  |
|  |  |  |  |
| Consensus discussions on guideline use and support helps me to  actually support practitioners with their guideline use. |  | Formal commitments on guideline use and support helps me to  actually support practitioners with their guideline use. | |
|  |  |  | |
| Consensus discussions on guideline use and support could help me to actually support profs with their guideline use |  | Formal commitments on guideline use and support could help me to actually support profs with their guideline use | |
|  |  |  | |
| Practitioners | | | |
|  |  |  | |
| I am experiencing management support on guideline use. | | | |
|  |  |  | |
| Management support on guideline use has … my actual guideline use. | | | |
|  | | | |
| Management support on guideline use could … my actual guideline use. | | | |

**Type B hypotheses**

| KNOWLEDGE ABOUT GUIDELINE USE | | |
| --- | --- | --- |
|  |  |  |
| Create a learning collaborative - Instructions how to perform the behavior |  | Conduct educational meetings - Instructions how to perform the behavior |
|  |  |  |
| Practitioners | | |
|  | | |
| Did you receive instructions on the guideline and its use? | | |
|  |  |  |
| I received specific instructions on guideline use  during collaborative learning sessions. |  | I received specific instructions on guideline use  during educational meetings. |
|  |  |  |
| Receiving specific instructions during collaborative learnings about guideline use helps me to increase my knowledge regarding guideline use. | | |
|  | | |
| Receiving specific instructions during collaborative learnings about guideline use could help me to increase my knowledge regarding guideline use. | | |
|  | | |
| Increasing my knowledge regarding guideline use has ... my actual guideline use. | | |
|  | | |
| Increasing my knowledge regarding guideline use could ... my actual guideline use. | | |

| COMMUNICATION SKILLS | | |
| --- | --- | --- |
|  |  |  |
| Conduct ongoing training - Behavioral practice/rehearsal |  | Conduct educational outreach visits - Behavioral practice/rehearsal |
|  |  |  |
| Practitioners | | |
|  | | |
| Did you have communication training to practice your communication skills? | | |
|  |  |  |
| How often did you have communication training? |  | Did you have communication training from a  Practitioner with expertise in communication skills? |
|  |  |  |
| Repeated training is of added value in practicing my communication skills. |  | Receiving communication training from an external professional helps me to practice my communication skills. |
|  | | |
| Repeated training could be of added value in practicing my communication skills. |  | Receiving communication training from an external professional could help me with practicing my communication skills. |
|  | | |
| Practicing communication skills helps me to improve my actual communication skills. | | |
|  | | |
| Practicing communication skills could me to improve my actual communication skills. | | |
|  | | |
| Improving my communication skills has ... my actual guideline use. | | |
|  | | |
| Improving my communication skills could ... my actual guideline use. | | |
