# Supplementary material for: Optimizing implementation: elucidating the role of behavior change techniques and corresponding strategies on determinants and implementation performance: a cross-sectional study
Source: Implement Sci Commun. 2024 Jun 20;5:68. doi: 10.1186/s43058-024-00604-w (PMC11191141; doi:10.1186/s43058-024-00604-w)
Supplement: Supplementary file 4 — Supplementary Material 4. [file 43058_2024_604_MOESM4_ESM.docx]

**Univariate logistic regression analysis for implementation determinants and self-reported implementation level**

| **Table A1.** Univariate logistic regression analysis for implementation determinants and self-reported implementation level. | | | | |
| --- | --- | --- | --- | --- |
| Determinants | | *N* (%) | OR | 95% CI |
| **Process of implementation** | |  |  |  |
|  | Guideline promotion [yes] | 92 (51.1) | **2.69** | 1.28-5.64 |
|  | Mandatory education [yes] | 72 (40.0) | 1.75 | 0.82-3.72 |
|  | Motivated implementation leader [yes] | 68 (37.8) | **3.64** | 1.51-8.79 |
|  | Management support [yes] | 101 (56.1) | **4.75** | 2.19-10.30 |
| **Knowledge and skills** | |  |  |  |
|  | Knowledge about guideline use [yes] | 123 (68.3) | **2.43** | 1.18-5.01 |
|  | Communication skills [yes] | 67 (37.2) | **2.44** | 1.08-5.50 |
| **Bold** = p<.05. | | | | |

**Univariate and multivariate logistic regression analysis for hypotheses type A2 and implementation performance**

| **Table A2.** Univariate and multivariate logistic regression analysis for perceived guideline promotion and implementation performance. | | | | | | | | |
| --- | --- | --- | --- | --- | --- | --- | --- | --- |
| Hypotheses | | *n (%)* | Univariate | | Multivariate | | | |
|  | |  |  | | Block 1 | | Block 2 | |
|  | |  | OR | 95% CI | OR | 95% CI | OR | 95% CI |
| Guideline promotion [yes] | |  | **2.69** | 1.28-5.64 | **2.69** | 1.28-5.64 | **2.91** | 1.30-6.51 |
|  | |  |  |  |  |  |  |  |
| Organization [(forensic) Mental health care] | | 149 (82.8) | 1.56 | 0.65-3.72 |  |  | 1.16 | 0.42-3.19 |
| Professional degree | |  |  |  |  |  |  |  |
|  | *Higher education* | 75 (41.7) | 1 |  |  |  | 1 |  |
|  | *University degree* | 31 (17.2) | 2.95 | 0.80-10.85 |  |  | **4.86** | 1.15-20.63 |
|  | *Master’s degree* | 62 (34.4) | 0.91 | 0.42-1.98 |  |  | 0.86 | 0.36-2.02 |
|  | *Specialized medical degree* | 12 (6.7) | 0.95 | 0.23-3.88 |  |  | 1.52 | 0.31-7.43 |
| Policy officer DV and CA | | 41 (22.8) | 1.51 | 0.61-3.73 |  |  | 1.30 | 0.46-3.65 |
| Work experience [in years]* | | 28.52 (7.27) | **1.06** | 1.01-1.11 |  |  | **1.06** | 1.00-1.12 |
| Working hours [per week]* | | 17.59 (9.92) | 1.01 | 0.97-1.04 |  |  | 1.01 | 0.96-1.05 |
| Guideline objective [DV and/or CA] | | 109 (60.6) | 1.99 | 0.98-4.05 |  |  | 1.26 | 0.54-2.96 |
| Nagelkerke R^2^ | |  |  |  | 0.06 |  | 0.16 |  |
| * mean(SD); DV=Domestic Violence; CA=Child Abuse; OR=Odds Ratio; **bold**=p<.05. | | | | | | | | |

| **Table A3.** Univariate and multivariate logistic regression analysis for the presence of a motivated implementation leader and implementation performance. | | | | | | | | |
| --- | --- | --- | --- | --- | --- | --- | --- | --- |
| Hypotheses | | *n (%)* | Univariate | | Multivariate | | | |
|  | |  |  | | Block 1 | | Block 2 | |
|  | |  | OR | 95% CI | OR | 95% CI | OR | 95% CI |
| Motivated implementation leader [yes] | | 94 (52.2) | **3.64** | 1.51-8.79 | **3.64** | 1.51-8.79 | **4.44** | 1.67-11.84 |
|  | |  |  |  |  |  |  |  |
| Organization [(forensic) Mental health care] | | 149 (82.8) | 1.56 | 0.65-3.72 |  |  | 1.02 | 0.38-2.78 |
| Professional degree | |  |  |  |  |  |  |  |
|  | *Higher education* | 75 (41.7) | 1 |  |  |  | 1 |  |
|  | *University degree* | 31 (17.2) | 2.95 | 0.80-10.85 |  |  | **4.68** | 1.11-19.90 |
|  | *Master’s degree* | 62 (34.4) | 0.91 | 0.42-1.98 |  |  | 0.86 | 0.36-2.06 |
|  | *Specialized medical degree* | 12 (6.7) | 0.95 | 0.23-3.88 |  |  | 2.10 | 0.42-10.51 |
| Policy officer DV and CA | | 41 (22.8) | 1.51 | 0.61-3.73 |  |  | 1.23 | 0.42-3.61 |
| Work experience [in years]* | | 28.52 (7.27) | **1.06** | 1.01-1.11 |  |  | **1.08** | 1.01-1.14 |
| Working hours [per week]* | | 17.59 (9.92) | 1.01 | 0.97-1.04 |  |  | 0.99 | 0.94-1.04 |
| Guideline objective [DV and/or CA] | | 109 (60.6) | 1.99 | 0.98-4.05 |  |  | 1.22 | 0.52-2.83 |
| Nagelkerke R^2^ | |  |  |  | 0.08 |  | 0.19 |  |
| * mean(SD); DV=Domestic Violence; CA=Child Abuse; OR=Odds Ratio; **bold**=p<.05. | | | | | | | | |

| **Table A4.** Univariate and multivariate logistic regression analysis for perceived management support and implementation performance. | | | | | | | | |
| --- | --- | --- | --- | --- | --- | --- | --- | --- |
| Hypotheses | | *n (%)* | Univariate | | Multivariate | | | |
|  | |  |  | | Block 1 | | Block 2 | |
|  | |  | OR | 95% CI | OR | 95% CI | OR | 95% CI |
| Management support [yes] | | 94 (52.2) | **4.75** | 2.19-10.10 | **4.75** | 2.19-10.10 | **4.44** | 1.95-10.11 |
|  | |  |  |  |  |  |  |  |
| Organization [(forensic) Mental health care] | | 149 (82.8) | 1.56 | 0.65-3.72 |  |  | 1.21 | 0.43-3.35 |
| Professional degree | |  |  |  |  |  |  |  |
|  | *Higher education* | 75 (41.7) | 1 |  |  |  | 1 |  |
|  | *University degree* | 31 (17.2) | 2.95 | 0.80-10.85 |  |  | **3.99** | 0.93-17.24 |
|  | *Master’s degree* | 62 (34.4) | 0.91 | 0.42-1.98 |  |  | 0.83 | 0.34-1.99 |
|  | *Specialized medical degree* | 12 (6.7) | 0.95 | 0.23-3.88 |  |  | 1.70 | 0.35-8.32 |
| Policy officer DV and CA | | 41 (22.8) | 1.51 | 0.61-3.73 |  |  | 1.45 | 0.50-4.19 |
| Work experience [in years]* | | 28.52 (7.27) | **1.06** | 1.01-1.11 |  |  | 1.05 | 0.99-1.11 |
| Working hours [per week]* | | 17.59 (9.92) | 1.01 | 0.97-1.04 |  |  | 1.01 | 0.96-1.06 |
| Guideline objective [DV and/or CA] | | 109 (60.6) | 1.99 | 0.98-4.05 |  |  | 1.24 | 0.52-2.94 |
| Nagelkerke R^2^ | |  |  |  | 0.14 |  | 0.21 |  |
| * mean(SD); DV=Domestic Violence; CA=Child Abuse; OR=Odds Ratio; **bold**=p<.05. | | | | | | | | |

| **Table A5.** Univariate and multivariate logistic regression analysis for knowledge about guideline use (hypothesis 1) and implementation performance. | | | | | | | | |
| --- | --- | --- | --- | --- | --- | --- | --- | --- |
| Hypotheses | | *n (%)* | Univariate | | Multivariate | | | |
|  | |  |  | | Block 1 | | Block 2 | |
|  | |  | OR | 95% CI | OR | 95% CI | OR | 95% CI |
| Instructions on how to perform a behaviour – create a learning collaborative | |  |  |  |  |  |  |  |
|  | *Part 2 present [yes]* | 94 (52.2) | **2.16** | 1.05-4.44 | **2.21** | 1.05-4.44 | 2.19 | 1.00-4.77 |
|  | |  |  |  |  |  |  |  |
| Organization [(forensic) Mental health care] | | 149 (82.8) | 1.56 | 0.65-3.72 |  |  | 1.27 |  |
| Professional degree | |  |  |  |  |  |  |  |
|  | *Higher education* | 75 (41.7) | 1 |  |  |  | 1 |  |
|  | *University degree* | 31 (17.2) | 2.95 | 0.80-10.85 |  |  | **4.39** | 1.05-18.27 |
|  | *Master’s degree* | 62 (34.4) | 0.91 | 0.42-1.98 |  |  | 0.92 | 0.40-2.16 |
|  | *Specialized medical degree* | 12 (6.7) | 0.95 | 0.23-3.88 |  |  | 1.76 | 0.36-8.49 |
| Policy officer DV and CA | | 41 (22.8) | 1.51 | 0.61-3.73 |  |  | 1.17 | 0.43-3.23 |
| Work experience [in years]* | | 28.52 (7.27) | **1.06** | 1.01-1.11 |  |  | 1.05 | 1.00-1.12 |
| Working hours [per week]* | | 17.59 (9.92) | 1.01 | 0.97-1.04 |  |  | 1.00 | 0.96-1.05 |
| Guideline objective [DV and/or CA] | | 109 (60.6) | 1.99 | 0.98-4.05 |  |  | 1.54 | 0.68-3.49 |
| Nagelkerke R^2^ | |  |  |  | 0.04 |  | 0.14 |  |
| * mean(SD); DV=Domestic Violence; CA=Child Abuse; OR=Odds Ratio; **bold**=p<.05. | | | | | | | | |

**Univariate and multivariate logistic regression analysis for hypotheses type B and implementation performance**

| **Table A6.** Univariate and multivariate logistic regression analysis for knowledge about guideline use (hypothesis 2) and implementation performance. | | | | | | | | |
| --- | --- | --- | --- | --- | --- | --- | --- | --- |
| Hypotheses | | *n (%)* | Univariate | | Multivariate | | | |
|  | |  |  | | Block 1 | | Block 2 | |
|  | |  | OR | 95% CI | OR | 95% CI | OR | 95% CI |
| Instructions on how to perform a behaviour – conduct educational meetings | |  |  |  |  |  |  |  |
|  | *Part 2 present [yes]* | 114 (63.3) | **2.33** | 1.14-4.77 | **2.33** | 1.14-4.77 | **2.22** | 1.03-4.79 |
|  | |  |  |  |  |  |  |  |
| Organization [(forensic) Mental health care] | | 149 (82.8) | 1.56 | 0.65-3.72 |  |  | 1.18 | 0.43-3.24 |
| Professional degree | |  |  |  |  |  |  |  |
|  | *Higher education* | 75 (41.7) | 1 |  |  |  | 1 |  |
|  | *University degree* | 31 (17.2) | 2.95 | 0.80-10.85 |  |  | 3.72 | 0.90-15.34 |
|  | *Master’s degree* | 62 (34.4) | 0.91 | 0.42-1.98 |  |  | 0.95 | 0.40-2.25 |
|  | *Specialized medical degree* | 12 (6.7) | 0.95 | 0.23-3.88 |  |  | 1.45 | 0.31-6.67 |
| Policy officer DV and CA | | 41 (22.8) | 1.51 | 0.61-3.73 |  |  | 1.11 | 0.40-3.05 |
| Work experience [in years]* | | 28.52 (7.27) | **1.06** | 1.01-1.11 |  |  | **1.06** | 1.00-1.13 |
| Working hours [per week]* | | 17.59 (9.92) | 1.01 | 0.97-1.04 |  |  | 1.01 | 0.96-1.05 |
| Guideline objective [DV and/or CA] | | 109 (60.6) | 1.99 | 0.98-4.05 |  |  | 1.40 | 0.61-3.22 |
| Nagelkerke R^2^ | |  |  |  | 0.05 |  | 0.14 |  |
| * mean(SD); DV=Domestic Violence; CA=Child Abuse; OR=Odds Ratio; **bold**=p<.05. | | | | | | | | |

| **Table A7.** Univariate and multivariate logistic regression analysis for communication skills (hypothesis 1) and implementation performance. | | | | | | | | |
| --- | --- | --- | --- | --- | --- | --- | --- | --- |
| Hypotheses | | *n (%)* | Univariate | | Multivariate | | | |
|  | |  |  | | Block 1 | | Block 2 | |
|  | |  | OR | 95% CI | OR | 95% CI | OR | 95% CI |
| Behavioral practice/rehearsal – conduct educational outreach visits | |  |  |  |  |  |  |  |
|  | *Part 2 present [yes]* | 43 (29.4) | 2.31 | 0.95-5.61 | 2.31 | 0.95-5.61 | 2.13 | 0.83-5.47 |
|  | |  |  |  |  |  |  |  |
| Organization [(forensic) Mental health care] | | 149 (82.8) | 1.56 | 0.65-3.72 |  |  | 1.23 | 0.44-3.41 |
| Professional degree | |  |  |  |  |  |  |  |
|  | *Higher education* | 75 (41.7) | 1 |  |  |  | 1 |  |
|  | *University degree* | 31 (17.2) | 2.95 | 0.80-10.85 |  |  | 4.12 | 0.99-17.10 |
|  | *Master’s degree* | 62 (34.4) | 0.91 | 0.42-1.98 |  |  | 0.93 | 0.39-2.20 |
|  | *Specialized medical degree* | 12 (6.7) | 0.95 | 0.23-3.88 |  |  | 1.75 | 0.37-8.18 |
| Policy officer DV and CA [yes] | | 41 (22.8) | 1.51 | 0.61-3.73 |  |  | 1.15 | 0.42-3.20 |
| Work experience [in years] | | 28.52 (7.27) | **1.06** | 1.01-1.11 |  |  | 1.05 | 1.00-1.12 |
| Working hours [per week] | | 17.59 (9.92) | 1.01 | 0.97-1.04 |  |  | 1.00 | 0.96-1.05 |
| Guideline objective [DV and/or CA] | | 109 (60.6) | 1.99 | 0.98-4.05 |  |  | 1.48 | 0.65-3.41 |
| Nagelkerke R^2^ | |  |  |  | 0.03 |  | 0.13 |  |
| * mean(SD); DV=Domestic Violence; CA=Child Abuse; OR=Odds Ratio; **bold**=p<.05. | | | | | | | | |

| **Table A8.** Univariate and multivariate logistic regression analysis for communication skills (hypothesis 2) and implementation performance. | | | | | | | | |
| --- | --- | --- | --- | --- | --- | --- | --- | --- |
| Hypotheses | | *n (%)* | Univariate | | Multivariate | | | |
|  | |  |  | | Block 1 | | Block 2 | |
|  | |  | OR | 95% CI | OR | 95% CI | OR | 95% CI |
| Behavioral practice/rehearsal – conduct ongoing training | |  |  |  |  |  |  |  |
|  | *Part 2 present [yes]* | 53 (29.4) | 2.31 | 0.95-5.61 | 2.31 | 0.95-5.61 | **2.80** |  |
|  | |  |  |  |  |  |  |  |
| Organization [(forensic) Mental health care] | | 149 (82.8) | 1.56 | 0.65-3.72 |  |  | 1.18 |  |
| Professional degree | |  |  |  |  |  |  |  |
|  | *Higher education* | 75 (41.7) | 1 |  |  |  |  |  |
|  | *University degree* | 31 (17.2) | 2.95 | 0.80-10.85 |  |  | **5.43** | 1.27-23.29 |
|  | *Master’s degree* | 62 (34.4) | 0.91 | 0.42-1.98 |  |  | 1.04 | 0.44-2.49 |
|  | *Specialized medical degree* | 12 (6.7) | 0.95 | 0.23-3.88 |  |  | 2.07 | 0.43-9.94 |
| Policy officer DV and CA [yes] | | 41 (22.8) | 1.51 | 0.61-3.73 |  |  | 1.19 | 0.42-3.36 |
| Work experience [in years] | | 28.52 (7.27) | **1.06** | 1.01-1.11 |  |  | 1.06 | 1.00-1.12 |
| Working hours [per week] | | 17.59 (9.92) | 1.01 | 0.97-1.04 |  |  | 1.01 | 0.96-1.06 |
| Guideline objective [DV and/or CA] | | 109 (60.6) | 1.99 | 0.98-4.05 |  |  | 1.59 | 0.69-3.63 |
| Nagelkerke R^2^ | |  |  |  | 0.03 |  | 0.15 |  |
| * mean(SD); DV=Domestic Violence; CA=Child Abuse; OR=Odds Ratio; **bold**=p<.05. | | | | | | | | |
